# Targeting Multiple-Myeloma-Induced Immune Dysfunction to Improve Immunotherapy Outcomes

**DOI:** 10.1155/2012/196063

**Published:** 2012-04-11

**Authors:** Sergio Rutella, Franco Locatelli

**Affiliations:** ^1^Department of Pediatric Hematology/Oncology, IRCCS Bambino Gesù Children's Hospital, Piazza Sant'Onofrio 4, 00165 Rome, Italy; ^2^Catholic University Medical School, 00168 Rome, Italy; ^3^University of Pavia, 27100 Pavia, Italy

## Abstract

Multiple myeloma (MM) is a plasma cell malignancy associated with high levels of monoclonal (M) protein in the blood and/or serum. MM can occur *de novo* or evolve from benign monoclonal gammopathy of undetermined significance (MGUS). Current translational research into MM focuses on the development of combination therapies directed against molecularly defined targets and that are aimed at achieving durable clinical responses. MM cells have a unique ability to evade immunosurveillance through several mechanisms including, among others, expansion of regulatory T cells (Treg), reduced T-cell cytotoxic activity and responsiveness to IL-2, defects in B-cell immunity, and induction of dendritic cell (DC) dysfunction. Immune defects could be a major cause of failure of the recent immunotherapy trials in MM. This article summarizes our current knowledge on the molecular determinants of immune evasion in patients with MM and highlights how these pathways can be targeted to improve patients' clinical outcome.

## 1. Introduction

Multiple myeloma (MM) is a malignant plasma cell (PC) disorder, accounting for approximately 1% of neoplastic diseases and 13% of all hematological cancers [1]. It may either present *de novo* or evolve from a monoclonal gammopathy of undetermined significance (MGUS) that progresses to smoldering myeloma (SMM) and, finally, to symptomatic MM. In recent years, the introduction of autologous hematopoietic stem cell transplantation (HSCT) and the availability of novel drugs such as thalidomide, lenalidomide, and bortezomib, have prolonged overall survival [1]. Patients with standard risk factors (absence of t(4; 14), t(14; 16), 17p-) are expected to live for 7 to 10 years from diagnosis, with good quality of life. In spite of these developments, MM remains an incurable disease for the vast majority of patients.

MM tumor cells are susceptible to immune recognition, as suggested by the therapeutic efficacy of allogeneic HSCT in patients with this disease [2]. In fact, the curative potential of allogeneic HSCT has been attributed, at least in large part, to the graft-versus-myeloma effect, at best illustrated by the induction of sustained molecular remissions after donor lymphocyte infusions (DLIs) in patients with either relapsed or persistent disease after allogeneic HSCT [3]. Moreover, in 357 cases of MM, event-free and overall survival were improved in patients given autologous-allogeneic HSCT (tandem transplantation) as compared with patients lacking an HLA-matched sibling donor and receiving double-autologous HSCT [4]. It should be mentioned that another study recruiting 710 myeloma patients with both standard-risk and high-risk disease from 37 transplant centers across the United States failed to show any superiority of nonmyeloablative (NMA) allogeneic HSCT after autologous HSCT compared with tandem autologous HSCT in terms of 3-year progression-free and overall survival [5], suggesting that strategies aimed at enhancing the antimyeloma effect are needed to improve the outcome of NMA transplants.

Despite these data indicating that an alloreactive-mediated, graft-versus-myeloma (GVM) effect may be crucial for tumor eradication, MM is unique in its ability to elude immunosurveillance, as a result of qualitative and/or quantitative abnormalities of DC and Treg cells, and of enhanced release of immunoregulatory cytokines by microenvironmental cells. Example of the latter mechanism is secretion, by bone marrow (BM) stromal cells (BMSCs), of immunomodulatory and proangiogenic growth factors, such as transforming growth factor (TGF)-*β*, vascular endothelial growth factor, interleukin (IL)-6, and hepatocyte growth factor (HGF). This article will review our current knowledge on the mechanisms whereby MM cells downregulate/improve antimyeloma immunity and will discuss how these circuits can be targeted to improve treatment outcomes.

## 2. Evidence for an Immune-Mediated Graft-versus-Myeloma Effect

As previously mentioned, DLI can induce remissions in patients with either relapsed or persistent MM after allogeneic HSCT. Responses to DLI and chronic GVHD are closely correlated, indicating that cellular/molecular targets for GVHD and GVM are similar or identical and may include the minor histocompatibility antigens (mHa) expressed on both normal and MM cells of the patients. Limited chronic GVHD has been correlated with a significantly decreased risk of myeloma recurrence and with longer event-free survival after NMA and reduced-intensity conditioning (RIC) transplants, consistent with a beneficial effect of the GVM response [6]. The European Group for Blood and Marrow Transplantation (EBMT) has published the results obtained in 229 MM patients who received an allogeneic HSCT with RIC regimens from 33 clinical centres [7]. Chronic GVHD was associated with better overall survival and progression-free survival that were 84% and 46% for patients with limited chronic GVHD, 58% and 30% for those with extensive chronic GVHD, and 29% and 12% in the absence of chronic GVHD, respectively, again underscoring the importance of the GVM effect [7].

Support for the role of mHa antigens in the GMV effect is provided by the experimental observation that malignant plasma cells may be susceptible to HA1-specific lysis *in vitro* [8]. Immune modulating drugs, such as thalidomide, administered after allogeneic HSCT, might be of therapeutic interest. Thalidomide, given at low doses and, after an interval of 14 days, followed by DLI, has been reported to improve responses to DLI, without inducing GVHD [9]. In a series of 18 patients so treated, 2 developed acute GVHD grade I of the skin, and only 2 developed *de novo* chronic GVHD. The high response rate of 67% with 22% complete remission indicated an additive or synergistic antimyeloma effect.

The cancer testis (CT) antigen class of tumor antigens is also a potential target for the GVM effect. MAGE-type genes can be detected in the majority of MM patients with advanced disease, but not in samples from patients with MGUS and with stage I/II myeloma [10]. CT10/MAGEC2, MAGEA3, BAGE, and NY-ESO-1 mRNA have been detected in about 90% of MM cell lines [11]. MAGEC2 and MAGEC3 were the most frequently expressed CT antigens in a cohort of 55 patients with advanced MM. Furthermore, IgG antibodies towards CT antigens were detected in the serum of 10 out of 66 analyzed samples. Nine out of 10 patients with detectable antibody responses had undergone allogeneic HSCT. When paired analyses were performed with sera collected before and after allogeneic HSCT in 7 out of 9 allotransplanted patients, none of these patients showed antibody responses against any of the CT antigens in their pretransplantation sample. The CT antigen NY-ESO-1 also elicited strong allogeneic T-cell responses in one patient with MM who received allogeneic HSCT [11]. Through the use of 12mer peptides overlapping by a single amino acid and spanning NY-ESO-1 region 51–70, it was shown that the CD4^+^ and CD8^+^ T-cell responses were both directed against NY-ESO-1_51–62_. When investigating the HLA restriction patterns of T-cell responses against NY-ESO-1_51–62_, only HLA-DQ5-expressing EBV-B cell lines were capable of presenting NY-ESO-1_51–62  _ to the CD4^+^ T cells, whereas the CD8^+^ response against the same peptide was restricted by HLA-B27. The preferential expression of NY-ESO-1 has also been described in MM patients with cytogenetic abnormalities [12]. In this study, spontaneous antibody responses to NY-ESO-1 could be demonstrated in 33% of NY-ESO-1^+^ MM patients. Furthermore, NY-ESO-1_157–165_-specific T cells, accounting for 0.2–0.6% of CD8^+^ T cells, were detected in NY-ESO-1^+^ MM patients with HLA-A*0201 tetramers.

Altogether, there is strong evidence to support that immune responses against myeloma are crucial for disease control. This implies that strategies aimed at counteracting the myeloma-induced immune dysfunction should be able to translate into better outcomes.

## 3. Immune-Suppressive Molecules Expressed by MM Cells

The outcome of an immune response is dependent on the multiple signals exchanged by antigen-presenting cells (APCs) and antigen-specific T cells, and on the provision of cytokines and membrane-bound costimulatory molecules, especially those of the B7-CD28 family [13]. The classical B7-CD28 pathway includes 2 ligands, B7.1/CD80 and B7.2/CD86 on cell surface of APC and at least 2 receptors, CD28 and cytotoxic T-lymphocyte antigen 4 (CTLA-4), on T cells. The interaction between CD80/CD86 ligands on APC and CD28/CTLA-4 on T cells controls antigen-specific T-cell proliferation, anergy, and survival. More recently identified B7-homologs, including B7-H1/programmed death-ligand 1 (PD-L1), B7-DC/PD-L2, B7-H2/inducible costimulator ligand (ICOSL)/B7h/B7RP-1, are expressed on APC as well as on cells within nonlymphoid organs. Both PD-L1 and PD-L2 interact with PD-1, a member of the CD28 family, whereas ICOSL is known to bind to ICOS. The expression of CD86 and ICOSL in acute myeloid leukemia has been correlated with inhibition of antitumor immunity and with a poor prognosis [14].

A survey of murine tumor lines has revealed that myeloma cell lines naturally express PD-L1 [15]. Growth of myeloma cells in normal syngeneic mice was inhibited significantly, albeit transiently, by administration of anti-PD-L1 antibody *in vivo* and was suppressed completely in the syngeneic PD-1-deficient mice [15]. The expression of PD-L1 by MM cell lines may also be associated with reduced susceptibility to tumor cell lysis by cytotoxic T-lymphocyte (CTL) clones [16]. Among a series of cytokines tested, IFN-*γ* alone was capable of upregulating PD-L1 expression on patient-derived MM cells, mainly through the MEK/ERK signalling pathway [16]. PD-L1 was not detected in patients with MGUS or in healthy controls. Another study showed that PD-L1 is expressed by a subset of cycling CD34^+^CD138^+^ malignant PC [17]. Interestingly, PD-L1 participates in the induction and maintenance of Treg cells, in synergy with TGF-*β* [18]. In a mouse model of oral tolerance induced by the intragastric administration of chicken ovoalbumin, both PD-L1 and PD-L2 expressed on mesenteric lymph node DC contribute to the promotion of Treg differentiation [19].

In contrast to PD-L1, ICOSL is expressed by <10% of MM cases and is induced by TNF-*α* and/or autologous BMSCs [20]. MM cell lines and fresh myeloma samples also express CD86 [20]. When patients were dichotomized based on CD86 expression levels, all patients assigned to the CD86^high^ group had lower hemoglobin and platelet levels, although there were no differences in overall survival between groups [20]. ICOSL was detected only in 3 out of 35 patients examined, who had either chemotherapy-resistant disease (2 patients) or plasma cell leukemia (1 patient). Interestingly, it was shown that both autologous BMSC and exogenous TNF-*α* upregulate CD86 or ICOSL expression in >50% of patients examined. Both CD86 and ICOSL molecules enhanced the production of IL-10 by T cells that were cultured *in vitro* with MM cells [20]. It is tempting to speculate that T-cell-derived IL-10 may also affect antimyeloma responses in addition to promoting malignant plasma cell proliferation.

Malignant plasma cells in MM express syndecan-1 (CD138). However, clonogenic or “cancer stem cells” may be enriched in a fraction of CD138^−^ cells that express intranuclear Sry-HMG-box 2 (SOX2), an embryonic stem cell marker implicated in self-renewal and pluripotency [21]. IgG antibodies to SOX2 of both *κ* and *λ* chain specificity can be detected in patients with MGUS, but not in those with either SMM or MM. After stimulation with a library of overlapping 15-mer peptides spanning the entire SOX2 protein, SOX2-specific T cells could be detected in 11 out of 16 MGUS patients and included both CD4^+^ and CD8^+^ T cells. Conversely, SOX2-specific T-cells could not be detected from MM patients or healthy donors, even after repeated antigen stimulation rounds. The SOX2-specific T-cells were of the Th1 phenotype, as they produced IFN-*γ* but not IL-4 or IL-10 upon SOX2 challenge [21]. Importantly, prestimulation of BM mononuclear cells from MGUS patients with the SOX2 peptide library is translated into the inhibition of clonogenic growth, suggesting that targeting SOX2 immunity may restrain tumor expansion. Finally, with a median followup of 24 months, MGUS patients with anti-SOX2 T cells had a significantly lower likelihood of disease progression, with a 2-year progression-free survival rate of 100% versus 30% in MGUS patients lacking anti-SOX2 T cells [21]. This study thus underlines that the immune system has the ability to spontaneously recognize antigens in the preneoplastic stage of human cancer and that the pattern of antigens targeted by the T cells in preneoplastic lesions may differ from that in clinical cancer.

**Figure 1:**
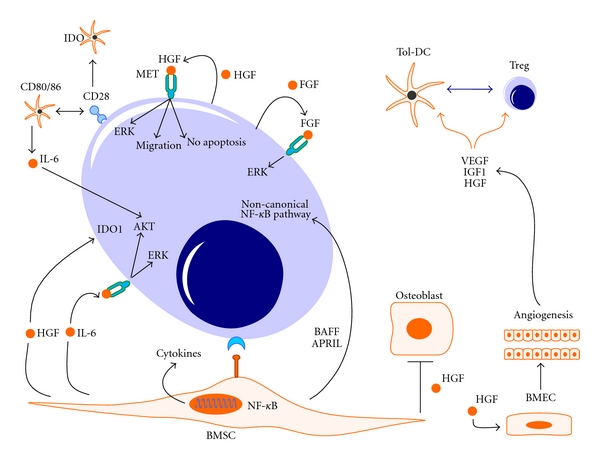
Interactions between myeloma and microenvironmental cell types. It is widely accepted that the BM microenvironment promotes myeloma growth [23]. Several cytokines can be released upon the interaction of MM plasma cells and BM microenvironmental cells, such as BM stromal cells (BMSCs), BM endothelial cells (BMECs), and osteoblasts. Among them, HGF is an attractive target for therapy, given its undisputed role in disease pathogenesis and its potential contribution to the myeloma-induced immune dysfunction through the upregulation of (IDO1) in MM cells. Insulin-like growth factor- (IGF-)1 receptor is also aberrantly expressed by myeloma cells and it has been associated with a poor prognosis [24]. The activation of cytokine networks ultimately leads to the development of immune suppression, through effects on Treg cells and DC. For instance, HGF has been shown to inhibit DC function both in mice and in humans [25, 26], favoring the emergence of tolerogenic DC. The main signaling pathways activated by HGF, IL-6, and other cytokines implicated in MM pathogenesis are indicated.

CD28 expression may be higher in malignant PC from patients with MM compared with SMM and healthy controls [22]. CD28 was mainly observed in patient subgroups with poor prognosis, as defined by the genetic signature. Importantly, myeloid DC that accumulate within the BM niche of patients with MM exert a prosurvival effect on CD28^+^ malignant PC that is mediated through CD28 interaction with CD80/CD86 on the DC. CD28-expressing MM cells induced IL-6 production upon coculture with CD80/CD86-expressing DC, a phenomenon that was markedly inhibited by blocking either the CD28-CD80/CD86 or Jagged-Notch-1 pathways [22]. Finally, the U266 MM cell line enhanced the IFN-*γ*-induced expression of IDO in DC, an effect that was also observed with primary myeloma cells and was largely CD28-dependent. This study suggests that CD28 expression by MM cells contributes to malignant cell survival and to the induction of an immune suppressive BM *milieu *(Figure 1).

CD200, formerly known as OX-2, is a highly conserved type I transmembrane glycoprotein that is expressed by thymocytes, activated T cells, B cells, DC, endothelial cells, and neurons. CD200 has been detected in malignant PCs of roughly 80% of patients with newly diagnosed MM [43]. In a group of 112 newly diagnosed patients treated with HSCT, patients with CD200^neg^ MM cells had a better event-free survival (24 months) compared with patients with CD200^pos^ MM cells (14 months) [43]. When CD200 expression was tested together with classical prognostic factors (serum albumin and serum *β*2-microglobulin), CD200 expression and *β*2-microglobulin remained independent prognostic factors [43]. The improved event-free survival of patients with MM cells lacking CD200 could be linked to the role of CD200 in suppression of T-cell-mediated immune responses and in the development of DC with a capacity to induce Treg cells [44].

## 4. Regulatory T-Cells in MM

Conflicting reports have been published on the frequency of Treg cells in patients with MGUS and MM, with studies showing either a decrease or an increase of FoxP3-expressing Treg cells [45–47]. The MM-specific idiotype immunoglobulin has been reported to expand Treg cells *in vitro* [48], although the specific epitope(s) or immunoglobulin domain(s) that are immunosuppressive in MM patients remain to be identified. Both Treg cells and myeloid-derived suppressor cells (MDSCs) were detected at increased frequency in a cohort of 76 patients with MM [46]. Treg cells were expanded only in patients at diagnosis, but not in those in remission or in patients with MGUS. The Treg cells were capable of inhibiting the proliferation of autologous responder CD4^+^ and CD8^+^ T cells by only 10% and 25%, respectively, suggesting that they may be dysfunctional. Another study conducted in 128 MM patients and 44 MGUS patients showed an increase of Treg cells, especially in the PB compared with the BM compartment [47]. The Treg cells from MM patients mediated similar levels of suppression of autologous T-cells compared with Treg cells from age-matched controls. Although Treg-cell numbers positively correlated with the paraprotein level, the highest numbers of Treg cells were identified in patients with low disease burden at the end of treatment in plateau phase response. In the newly diagnosed MM patients, there was a trend toward increased Treg numbers in the ISS stage II versus stage I. Finally, Treg numbers did not correlate with PB or BM levels of either IL-10 or TGF-*β*.

Another study involving 67 MM patients has shown that in both MGUS patients and untreated MM, as well as treated MM patients, the frequency of FoxP3-expressing T cells is increased compared with healthy controls [45]. Treg cells were isolated from 7 patients with untreated MM and were cocultured with allogeneic CD4^+^CD25^−^ T cells stimulated with irradiated PBMC as stimulators. On a percell basis, Treg from MM patients were equally effective at inhibiting allogeneic T-cell proliferation when compared with Treg cells from healthy controls. The inhibition of proliferation of conventional T cells by Treg cells from MM patients roughly equaled 60% when allogeneic T cells and Treg cells were cocultured at a (1 : 1) ratio. The same levels of suppression were observed when Treg cells from patients with MGUS were used in the coculture experiments.

Further analyses in 2 patients with MM suggested that Treg cells were mainly derived through the peripheral expansion of Treg cells, rather than through Treg generation within the thymus. Another study has shown that the frequency of FoxP3-expressing PBMC may be reduced in patients with MM or MGUS compared with healthy controls [49]. When cocultured with anti-CD3-activated PBMC, patient-derived Treg cells failed to suppress T-cell proliferation, even when added at a 10-fold higher number. It has been shown that the combined assessment of CD127 and FoxP3 expression may be superior to CD25/FoxP3 for an accurate identification of Treg cells in patients with cancer, including MM [50]. Collectively, these *ex vivo* studies suggest that Treg cells are abnormal in MM, either quantitatively or qualitatively. However, the biologic basis for Treg dysfunction in patients with MM and MGUS remains to be fully elucidated.

Importantly, Treg cells vigorously expand in the BM of MM patients given allogeneic HSCT [51]. At a median of 37 months from transplantation, BM-residing CD4^+^ T cells were markedly reduced compared with newly diagnosed MM patients and with healthy controls. Conversely, Treg cells were strongly enriched within the BM of transplanted patients, expressed TGF-*β* and CTLA-4, and exhibited full suppressor function against autologous non-Treg cells. The low number of T-cell excision circles (TRECs) documented in BM-resident Treg cells suggested that Treg cells were derived extrathymically, as a result of peripheral expansion. Finally, Treg cells preferentially expressed surface markers of naïve T cells, such as CD45RA. This study indicates that Treg-cell expansion after allogeneic HSCT may affect antimyeloma immunity and should be taken into consideration when designing adoptive immunotherapy approaches.

It should be emphasized that pharmacological agents active in MM, such as lenalidomide, thalidomide, and dexamethasone, may affect Treg numbers. Lenalidomide treatment in 8 patients with relapsed MM following allogeneic HSCT was associated with an increase of Treg numbers [52]. Conversely, lenalidomide may reduce the percentage of Treg cells in patients with chronic lymphocytic leukemia (CLL) [53, 54] and with solid tumors or myelodysplastic syndromes [55]. *In vitro*, lenalidomide inhibited Treg proliferation and diminished FoxP3 expression in the absence of measurable effects on TGF-*β* or IL-10 production, while reducing Treg cell accumulation in lymph nodes of CT26 cancer-bearing animals [56]. It thus remains to be determined whether lenalidomide may overcome the barriers to myeloma-specific immunity through the modulation of Treg cells also in humans. Similar to lenalidomide, thalidomide may diminish Treg numbers in patients with CLL [57], whereas it does not affect Treg function *in vitro* [56]. Further studies are needed to determine whether immune modulating drugs such as thalidomide and lenalidomide may affect Treg number and function in patients with MM, leading to a better immune control of the underlying disease.

## 5. BM Microenvironment and Immune Dysfunction in MM

The BM microenvironment encompasses a wide spectrum of cell types and extracellular matrix proteins, including fibronectin, collagen, laminin, and osteopontin [23]. Among the cell components, BMSC and bone marrow endothelial cells (BMECs) physically interact with MM cells and contribute to MM disease pathophysiology (Figure 1). BMSC further enhance the myeloma-induced immune dysfunction, by secreting factors such as VEGF, HGF, fibroblast growth factor (FGF), and stromal-cell-derived factor (SDF)-1*α*. The signalling pathways activated by the interaction between accessory cells and MM cells lead to growth, survival, and drug resistance of the latter, as well as to osteoclastogenesis and angiogenesis. For instance, the adhesion of MM cells to BMSC triggers the NF-*κ*B-dependent secretion of IL-6 in BMSC, further stimulating MM cell growth, survival and migration [58]. MM cells themselves secrete cytokines, such as TGF-*β* and VEGF, which promote IL-6 release from BMSC [59].

MM cells aberrantly express proangiogenic genes [60]. Although MM cells were shown not to express a significantly higher number of pro- or antiangiogenic genes compared with normal plasma cells, 97% of myeloma samples express at least one angiogenic factor among the 6 most frequently expressed factors, including HGF [61]. HGF is a 90-kd protein that signals through the MET receptor. Some MM cell lines and primary myeloma cells also secrete HGF, suggesting the occurrence of paracrine/autocrine interactions between microenvironmental cell types and myeloma cells *in vivo*. Stimulation of MM cells with HGF has been correlated with the activation of signalling pathways implicated in the regulation of cell proliferation and survival. Specifically, MEK is required for HGF-induced proliferation, whereas PI3K mediates myeloma cell rescue from apoptosis [62]. For biologic function, proteolytic conversion of single-chain HGF to the heterodimeric active form is essential. HGF activator (HGFA) is a factor XIIa-related serine protease secreted by the liver and that potently activates HGF. Intriguingly, HGFA levels are increased both in the PB and BM of patients with MM, providing a prerequisite for HGF activation *in vivo* [63]. Interestingly, myeloma cells catalyze HGF activation by secreting HGFA [64]. The processing of single-chain HGF was significantly enhanced by the addition of thrombin and was completely inhibited by serine protease inhibitors, such as aprotinin. Interestingly, single nucleotide polymorphisms of the *HGF* gene have been associated with myeloma risk [65].

High levels of HGF in serum and BM fluid of patients with MM predict a dismal prognosis, with a survival time of 32 and 21 months for patients with low and high HGF, respectively [66]. HGF values have been reported to decline after treatment with high-dose chemotherapy in patients with MM who obtain at least a partial response. Clinical responses to bortezomib and to high-dose chemotherapy have been shown to correlate with low pre-treatment concentrations of HGF [67, 68]. Conversely, pretreatment levels of other proangiogenic cytokines, such as VEGF and FGF, were not significantly different in responders versus non-responders. The Nordic Myeloma Study Group has shown that HGF is elevated in 25% of patients at diagnosis [69]. Following high-dose chemotherapy, median survival was not reached after 77 months in patients with normal HGF values, whereas in the group with elevated HGF, median survival was 63 months. Furthermore, HGF declined in a high proportion of MM patients at the time of disease remission.

Previously unappreciated effects of HGF on the immune response have been recently ascertained. In mice, treatment with HGF *in vitro* and *in vivo* suppresses the antigen-presenting function of DC [25]. The production of IL-12p70 is significantly inhibited by *in vivo* treatment with HGF, whereas IL-10 levels remain unaffected. HGF also inhibited antigen-induced T-cell activation in an indirect manner. Other studies showed that treatment with HGF ameliorates acute GVHD through effects on the proinflammatory cytokine cascades [70]. Serum IL-12 was significantly decreased in HGF-treated mice with GVHD and both IFN-*γ* and TNF-*α* were suppressed in target organs of GVHD, translating into a higher survival rate.

Indoleamine 2,3-dioxygenase 1 (IDO1) is a tryptophan-catabolizing enzyme and is constitutively expressed by a variety of human cancers, including acute myeloid leukemia [71, 72]. IDO1 expression in tumor cell lines and primary tumor cells is mainly triggered by IFN-*γ* and translates into tryptophan degradation into immune suppressive metabolites, collectively referred to as kynurenines [73]. We showed that HGF induces the expression of IDO1 in human-monocyte-derived DC [26] and that IDO1 may be expressed and functional in patients with MM, leading to Treg expansion [35]. Serum kynurenines correlated with HGF release, suggesting that HGF targeting should translate into restoration of antimyeloma immunity.

## 6. DC Dysfunction in MM

In mice, myeloma cells or tumor culture-conditioning medium (TCCM) inhibit the differentiation and function of DC, as shown by the lower expression of DC-related antigens and compromised capacity to activate allospecific T cells [74]. Treatment with TCCM activated p38 mitogen-activated protein kinase (MAPK) and Janus kinase (JNK) but inhibited extracellular regulated kinase (ERK). The inhibition of p38 MAPK restored the phenotype, cytokine secretion, and function of TCCM-treated DC, pointing to these alterations as novel mechanisms for tumor evasion that can be targeted to obtain more potent DC vaccines.

The absolute number of circulating precursors of myeloid and plasmacytoid DC may be significantly lower in MM patients than in healthy subjects [38]. In addition, patient-derived DC express significantly lower amounts of HLA-DR, CD40, and CD80 and are impaired in their ability to induce allogeneic T-cell proliferation. These phenotypic features closely resemble those assigned to tolerogenic DC populations [75]. The upregulation of CD80 on DC derived from MM patients is defective during stable disease and absent during progressive stages [38]. The inhibition of CD80 upregulation was reverted by blocking antibodies against TGF-*β* or IL-10. Although TGF-*β* and IL-10 are normal in most MM patients, cytoplasmic TGF-*β* was increased in plasma cells during progressive disease. Brown et al. [76] have further shown that DC numbers are only significantly decreased in patients with stage 3 disease. Both IL-12 and IFN-*γ* neutralize the failure to stimulate CD80 upregulation *in vitro*, suggesting that the addition of these cytokines to future immunotherapy trials should be considered. When investigating the effects of IL-6 on DC development and function, Ratta et al. [77] have demonstrated that IL-6 inhibits the colony growth of CD34^+^ DC progenitors and switches the commitment of CD34^+^ cells from DC to CD1a^−^CD14^+^ monocytic cells with potent phagocytic activity but without antigen-presenting function.

Inflammatory cytokines reportedly induce immunogenic DC suitable for immunotherapy. It has also been shown that human, monocyte-derived DCs matured in the presence of IL-1*β*, IL-6, and TNF-*α* expand functional Treg cells from patients with MM [78]. Importantly, the numbers of Treg cells may increase after the injection of cytokine-matured DC, as shown in 3 patients with MM and 1 patient with advanced renal cell cancer. The DC-mediated expansion of Treg cells was rapid, occurring as early as 7 days after the first DC injection, but was n

ot associated with clinical deterioration or decline in virus-specific immune responses. This study underscores a potentially detrimental role of vaccine-mediated induction of FoxP3^+^ Treg cells in patients with MM, a previously unappreciated effect in trials of human DC vaccination, and supports the need to combine DC therapy with approaches that selectively ablate Treg cells or inhibit their function.

## 7. Novel Strategies to Target Immune Suppressive Circuits in MM

CT-011 is a novel IgG1 humanized antibody that modulates the immune response through interaction with PD-1. MM cells express cognate ligands for PD-1, such as PD-L1. A phase I clinical trial of patients with advanced malignancies, including MM, has shown that CT-011 administration as a single intravenous dose is safe and well tolerated, with expansion of T-cell subsets and evidence of response in 33% of patients [79] (Table 1). CT-011 increases the migration of natural killer (NK) cells toward MM targets and enhances immune synapses between patient-derived NK cells and PD-L1-bearing, primary autologous MM cells [80]. CT-011 also increased NK-cell IFN-*γ* secretion against primary MM cells and enhanced NK cytotoxicity. Interestingly, lenalidomide downregulated PD-L1 expression on CD38^+^CD138^+^ primary MM tumor cells, independent of a direct apoptotic effect, suggesting that dual immunotherapy with CT-011 and lenalidomide may be justified in patients with MM. PD-L1 blockade has also been combined with syngeneic HSCT- and DC-based vaccination to improve outcomes in myeloma-bearing mice [81]. The PD-L1-expressing 5T33 tumor cell line was used to induce myeloma in mice. Interestingly, inhibition of the PD-1/PD-L1 pathway combined with HSCT and whole-cell vaccination increased the survival of myeloma-bearing mice from 0% to 40%. These results suggest that expression of PD-L1 can serve as a potent mechanism for potentially immunogenic tumors to escape from host immune responses, and that blockade of interaction between PD-1 and PD-L1 may offer a promising strategy for specific tumor immunotherapy.

It has been demonstrated that the MM immune tolerance can be overcome by modifying DC to express CNX, an accessory protein that enhances antigen processing and promotes DC and T-cell interactions [48]. CNX plays a key role in both major histocompatibility complex-class 1 and 2 antigen processing pathways and may also be involved in CD1d lipid antigen presentation [82]. Lentivirus-CNX-modified, myeloma DC effectively boosted cytokine production in both CD4^+^ and CD8^+^ T cells and increased cancer cell killing. These findings indicate that the tolerogenic DC in MM patients may be engineered into reactive DC to promote anticancer immunity with potential clinical benefit.

As discussed above, HGF is largely implicated in MM pathogenesis and is an intriguing target for antiangiogenesis and immunotherapy approaches (Table 1). MET-dependent invasive growth signals are currently viewed as a general feature of highly aggressive tumors. Molecules that inhibit MET and HGF can thus interfere with cancer onset and metastasis [29]. NK4, an antagonist for HGF, is

 composed of the NH_2_-terminal hairpin domain and 4 subsequent kringle domains of the *α*-subunit of HGF and is structurally similar to angiostatin. NK4 may exert antiangiogenic and tumor-suppressing activities independently of HGF antagonism. The expression of NK4 mediated by an adenovirus vector has been induced in mouse tumor cell lines [31]. The combination of NK4 with DC vaccination elicited synergistic antitumor effects. Tumor regression induced by NK4 and DC therapy required mainly MHC class I antigen presentation and T cells of the treated hosts [31]. Tumors in MHC class I-deficient mice lacking CD8^+^ T cells grew progressively, wherea

s MHC class 2-deficient mice responded to NK4 and DC vaccination with significant tumor suppression. Importantly, HGF antagonism translated into the emergence of antigen-specific CTL, as shown by the strong cytotoxic responses against parental B16-F10 melanoma cells and E.G7-OVA lymphoma cells achieved using splenocytes from tumor-bearing mice treated with NK4 and DC [31]. Interestingly, NK4 protein may stabilize the growth of MM cell lines and control the activation of MET, ERK1/2, STAT3, and AKT-1 [32]. When injected into myeloma-bearing mice, recombinant adenovirus containing NK4 cDNA inhibited myeloma growth, induced myeloma cell apoptosis, and restrained angiogenesis [32]. Although the effects of NK4 on antimyeloma immunity were not investigated, this study clearly indicates that molecular targeting of HGF by NK4 may prove beneficial in MM. Amgen has recently reported the generation of fully human monoclonal antibodies against HGF that exhibit therapeutic potential in mice bearing subcutaneous xenografts of human glioma cell lines with an HGF-dependent autocrine loop [27, 83, 84]. In particular, AMG102 (rilotumumab; Amgen) is a fully human neutralizing mAb against HGF and is currently under evaluation in patients with advanced solid tumors, both as monotherapy and in combination with other agents [83, 84]. The systemic administration of L2G7, another anti-HGF antibody from Galaxy Biotech, translated into the induction of regressions of glioma xenografts [28]. *In vivo* inhibition of glioblastoma growth also occurred with 5D5, a one-armed antibody against MET [30].

Finally, IDO1 inhibitors such as 1-methyl-tryptophan (1MT) have entered the clinical arena and have shown tolerability and induction of autoimmune responses in patients with solid tumors [36].

IDO1 may be located downstream of cyclooxygenase-2 (COX-2), as shown by the downregulation of IDO1 activity by COX-2 inhibitors [85, 86]. It has been shown that COX-2 expression by malignant PC confers an unfavorable prognosis to MM, being found in roughly 30% of newly diagnosed and 50% of relapsed/refractory MM cases [87]. In experimental pancreatic adenocarcinoma, the combined treatment with mucin-1- (MUC1-)based vaccine and celecoxib, a COX-2 inhibitor, elicited vigorous antitumor responses [88]. Mechanistically, the increased immune responses were correlated with the downregulation of circulating prostaglandin E2 and IDO enzymatic activities, leading to decreased levels of Treg cells within the tumor. This study strongly points to the COX-2/IDO1 interplay as a potential target for treatment also in MM.

### 7.1. Gene Modified T Cells

Human MM samples express ligands for NKG2D. Human T cells engineered to express chimeric NKG2D receptors consisting of NKG2D fused to the CD3*ζ* cytoplasmic domain lyse human myeloma cells [89]. The *in vivo* therapeutic efficacy of the chimeric T cells has been tested against an established 5T33 mouse model of myelom

a. Mice given chimeric T cells 5 and 12 days after myeloma inoculation experienced longterm survival compared with mice receiving wild-type NKG2D T cells [90]. The chimeric T cells could be found both in the spleen and in the BM of myeloma-bearing mice, although they did not survive long-term, being no longer detected 7 days af

ter T-cell injection. Interestingly, treatment with the chimeric T cells increased the activation of the host immune system, as reflected by the higher IFN-*γ* levels and CD69 expression in T-cell-treated, MM-bearing mice [90]. A chimeric T-cell receptor recognizing the carbohydrate antigen Lewis Y and containing CD3*ζ* and the CD28 coreceptor has been recently constructed [91]. Approximately 50% of primary myeloma samples expressed the Lewis Y antigen, which was not apparently related to any patient and clinical characteristics [91]. The transduced anti-Lewis T cells secreted IFN-*γ* in response to a myeloma cell line and specifically lysed Lewis^+^ myeloma targets. In addition, myeloma-bearing NOD/SCID mice received four intravenous injections of either chimeric T cells on days 0 (tumor challenge) and 1, 2, and 5, which translated into a significant improvement of survival compared with mice adoptively transferred with nontransduced T cells [91].

### 7.2. DC- and Id-Based Tumor Vaccines

DC-bases vaccination strategies have been explored to stimulate antimyeloma immune responses. The *ex vivo* generation of functionally active DC populations for adoptive transfer may circumvent the quantitative and qualitative disturbances of DC function in cancer patients. Tumor cells have been fused with autologous DC using polyethylene glycol, thus allowing the presentation of a broad array of antigens in the context of potent DC fusion partners. Bone-marrow-derived MM cells and patient-derived DC were used to generate DC/tumor fusions for a phase I clinical trial in 18 patients with active MM who had received at least 4 prior chemotherapy regimens [39]. The DC/tumor fusion cells were further activated with GM-CSF in culture prior to adoptive transfer. In 17 out of 18 patients, adequate numbers of DC/tumor fusions were obtained. In 11 out of 15 evaluable patients, vaccination elicited a 2-fold increase of CD4^+^ and CD8^+^ T cells reactive against a tumor lysate [39]. Humoral responses to vaccination also occurred, as suggested by the detection of antibodies against regulators of G-protein signaling 19 (RGS19), heat shock protein 90 (HSP90), and BRCA1-associated protein (BRAP). Myeloma disease was stabilized in 11 of the 16 evaluable patients for a variable period of time ranging from 2.5 (4 patients) to 41 months (1 patient) from vaccination [39].

DC vaccination has been pursued in patients who received an autologous HSCT, using the idiotype (Id) determinants on the MM immunoglobulin as tumor-specific antigens. Twenty-six patients with MM were enrolled in a phase I study of DC-based vaccination after autologous HSCT [92]. DCs were pulsed with the patient-derived Id chemically coupled with keyhole limpet hemocyanin (KLH). Of the 26 pati

ents, 17 were alive with a median follow-up of 34 months from transplantation. Anti-Id immune responses were documented in 4 patients, 3 of whom were in complete remission at the time of vaccination. The results of a phase II trial of Id-loaded APC, APC8020 (Mylovenge), given after autologous HSCTs for MM have been recently reported. Twenty-seven patients were enrolled on this trial and the outcomes were compared to that of 124 consecutive MM patients transplanted during the same period at Mayo Clinic [93]. After transplantation, 96% of patients in the vaccine trial and 88% of database patients achieved an objective response. Importantly, vaccinated patients had a significantly better overall survival (median: 5.3 years) compared with database patients (median: 3.4 years) [93]. The intranodal injection of Id-pulsed, CD40 ligand-matured DC has also been pursued in 9 patients with smoldering or stable myeloma disease [40]. Patients received low-dose IL-2 subcutaneously. At 5 years after vaccination, 5 patients had stable disease, whereas 4 patients had progressive disease. No modifications of either circulating T and B cells or BM plasma cells were observed compared with pre-treatment levels. In another study, immunization with the autologous myeloma Id-induced reductions of circulating clonal tumor B cells in patients with early-stage disease [41]. Id vaccination has also been applied to patients receiving high-dose chemotherapy followed by HSCT. Id vaccines and immune adjuvants were administered, together with either subcutaneous IL-2 or GM-CSF, to MM patients who were in disease remission at time of vaccination [94]. Delayed-type hypersensitivity (DTH) could be detected in 8 out of 10 patients, whereas Id-specific T-cell proliferative responses emerged in 2 out of 10 treated patients. Freedom from disease progression ranged from 9 to 36 months. Id-based DC vaccines were also given to MM patients 3 to 6 months after autologous HSCT [42]. Each patient in clinical stage III and with chemotherapy-responsive disease received 2 Id-pulsed DC vaccines, separated by 4 weeks. In 2 patients, Id-specific T-cell responses could be measured. With a minimum followup of 16 months from autologous HSCT and 3 months from DC-based vaccinations, 9 patients were alive [42]. In a subsequent phase I trial from the same authors, DC were obtained under serum-free conditions and were pulsed with patient-derived Id determinants [95]. Eight of 10 patients who received the scheduled vaccinations progressed, and 2 patients who were in partial response before vaccination remained in a clinically stable condition, with a followup of 25 and 20 months, respectively [95]. Id vaccines were combined with IL-12 or IL-12/GM-CSF administration in order to augment immune responses and improve clinical outcome [96]. In 1 out of 28 patients, a partial clinical response could be observed, whereas 11 out of 28 patients mounted T-cell proliferative responses to the Id, peaking within 8 weeks from the start of Id vaccination [96]. Collectively, DC- and Id-based immunotherapy trials have led to the emergence of antigen-specific immune responses in patients with MM, although clinical responses were detected in a minority of patients, underscoring the need for complementary strategies that overcome the myeloma-induced downregulation of immune responses.

Ipilimumab is a CTLA-4 antagonistic antibody. CTLA-4 is a T-cell-specific molecule that outcompetes CD28 and inhibits T-cell activation. In a phase III randomized, controlled trial in 676 patients with metastatic melanoma, treatment with ipilimumab improved the median overall survival by 3.7 months [97, 98]. It is tempting to speculate that CTLA-4 blockade may also restore immune responses against myeloma, either alone or in combination with vaccination strategy, as recently shown in patients with melanoma [99].

## 8. Concluding Remarks

MM has a unique ability to escape from immunosurveillance. The molecular determinants of immune suppression in MM can represent an ideal target to improve clinical outcome. The BM microenvironment is increasingly viewed as a crucial compartment where interactions between myeloma cells and stromal cells occur, leading to excessive plasma cell proliferation, survival, drug resistance, and migration capacity. Given the complexity of myeloma cell-microenvironmental interactions, combination therapies will be required to increase cytotoxicity and improve drug resistance [23]. The effects of novel immunomodulatory drugs on antimyeloma immunity remain to be thoroughly addressed. For instance, bortezomib has been reported to induce apoptosis in human monocyte-derived DC, but not in T cells or B cells [100]. Bortezomib may also impair the maturation of 6-sulfo LacNAc DC, a major subset of human blood DC, thus limiting the release of TNF-*α* and IL-12 [101]. It is presently unknown whether the down-regulation of DC function induced by bortezomib may adversely affect *in vivo* anti-myeloma immunity. A better understanding of the mechanisms underlying immune escape by myeloma cells will set the stage for clinical trials aimed at overcoming the immune system dysfunction associated with MM.

## Figures and Tables

**Table 1:** Immune suppressive circuits and molecular targets for immunotherapeutic approaches in MM. The mechanisms of immune evasion mediated by MM cells and the currently available strategies to target them are summarized.

Determinant(s) of immune dysfunction	Effect(s) on antimyeloma immune responses	Target(s) for intervention	Immunotherapeutic strategy	Phase of development (either pre-clinical or clinical)	Reference(s)
Secretion of proangiogenic cytokines within the MM microenvironment	-Induction of tolerogenic DC -Induction of IDO1		Anti-HGF antibodies	Not yet into the clinic for MM	[27, 28]
			MET inhibitors	Not yet into the clinic for MM	[29]
		HGF	Anti-MET antibodies	Not yet into the clinic for MM	[30]
			NK4 (HGF antagonist)	Not yet into the clinic	[31, 32]
		VEGF	Bevacizumab	Phase II, randomized	[33]

Expansion of CD25^+^Foxp3^+^ Treg cells	Inhibition of antimyeloma immunity	CD25	-Denileukin Diftitox (ONTAK) -CTLA4-Ig	Not yet into the clinic for MM	[34]

Enhanced tryptophan catabolism	Inhibition of antimyeloma immunity	IDO1	IDO1 chemical inhibitors	Not yet into the clinic for MM	[35, 36]

Expression of co-inhibitory receptors and other immune suppressive molecules	Expansion of Treg cells and inhibition of antimyeloma immunity	PD-L1	Anti-PD-1 antibodies (CT-011)	Pre-clinical	[15, 16]
		TGF-*β*	Anti-TGF-*β* antibodies	Not yet into the clinic	[37]
		IL-10	Anti-IL-10 antibodies	Not yet into the clinic	[38]

DC dysfunction	Inhibition of antimyeloma immunity	-MUC1 -Other MM antigens	DC/myeloma fusion cells	Phase I	[39]

Weak immunogenicity of MM-associated Id proteins	Weak antimyeloma immunity	Patients' idiotype	Id-based and DC-based vaccines	Phase I/II	[40–42]

Maintenance of clonogenic MM precursors	Unrestrained growth of MM cells	SOX2	Generation of SOX2-specific T cells with peptides spanning the SOX-2 protein	Not yet into the clinic	[21]
